# Retraction: Knockdown of NEAT1 ameliorated MPP^+^-induced neuronal damage by sponging miR-221 in SH-SY5Y cells

**DOI:** 10.1039/d1ra90056k

**Published:** 2021-02-03

**Authors:** Laura Fisher

**Affiliations:** Royal Society of Chemistry Thomas Graham House, Science Park, Milton Road Cambridge CB4 0WF UK advances-rsc@rsc.org

## Abstract

Retraction of ‘Knockdown of NEAT1 ameliorated MPP^+^-induced neuronal damage by sponging miR-221 in SH-SY5Y cells’ by Lijiao Geng *et al.*, *RSC Adv.*, 2019, **9**, 25257–25265, DOI: 10.1039/C9RA05039F.

The Royal Society of Chemistry hereby wholly retracts this *RSC Advances* article due to concerns with the reliability of the data. The images in the article, and the raw data provided by the authors, were screened by an image integrity expert. Based on the analysis of the raw data and comparison with the published images, the raw data bands may have been generated and then placed onto a false background. Therefore, the raw data provided by the authors cannot be used as verifiable raw data to validate the published images. Furthermore, the raw data provided by the authors was found to closely resemble raw data for a number of other articles, which is unexpected given that there are completely different author lists for these articles.

Given the significance of the concerns about the validity of both the data in the article and the raw data provided by the authors, the findings presented in this paper are not reliable.

Yong Chen does not agree to the retraction. The other authors have been informed but have not responded to any correspondence regarding the retraction.

Signed: Laura Fisher, Executive Editor, *RSC Advances*

Date: 19^th^ January 2021

## Supplementary Material

